# Prävalenz und diagnostische Aspekte von Gleichgewichtsstörungen bei Kindern mit Schwerhörigkeit

**DOI:** 10.1007/s00106-026-01728-2

**Published:** 2026-02-02

**Authors:** Julia Döge, Tim Watzlawik, Berit Hackenberg, Karl Melzer, Dzemal Gazibegovic, Andrea Bohnert, Kai Helling, Anne Läßig

**Affiliations:** 1 Hals‑, Nasen‑, Ohrenklinik und Poliklinik, Universitätsmedizin Mainz, Langenbeckstr. 1, 55131 Mainz, Deutschland; 2 Audiologisches Zentrum der Klinik und Poliklinik für Hals-Nasen-Ohren-Heilkunde, Universitätsklinikum Bonn, Bonn, Deutschland; 3 Abteilung Audiologische Akustik der Hals‑, Nasen‑, Ohrenklinik und Poliklinik, Universitätsmedizin Mainz, Mainz, Deutschland; 4 Schwerpunkt Kommunikationsstörungen der Hals-Nasen-Ohren-Klinik und Poliklinik, Universitätsmedizin Mainz, Mainz, Deutschland

**Keywords:** Vestibulär evozierte myogene Potenziale (VEMP), Neurootologische Diagnostik, Pädiatrische vestibuläre Störungen, Audiovestibuläre Störungen, Vestibuläre Dysfunktion, Vestibular evoked myogenic potentials (VEMP), Neurotology, Pediatric vestibular disorders, Audiovestibular disorders, Vestibular dysfunction

## Abstract

**Hintergrund:**

Kinder mit Schwerhörigkeit haben ein erhöhtes Risiko für vestibuläre Störungen. Aufgrund diagnostischer Herausforderungen wird eine entsprechende Untersuchung bei Kindern nur selten durchgeführt, sodass Daten zur Prävalenz in Deutschland weitgehend fehlen. Dabei könnten die Befunde Einfluss auf weitere Diagnostik, therapeutische Maßnahmen sowie Prävention haben.

**Ziel der Arbeit:**

Die vorliegende Arbeit untersucht die Machbarkeit einer Gleichgewichtsdiagnostik für Kinder aller Altersstufen und betont die Notwendigkeit ihrer routinemäßigen Anwendung bei Kindern mit Schwerhörigkeit. Zudem wurde die Prävalenz von Gleichgewichtsstörungen in dieser Population erfasst.

**Material und Methoden:**

Es wurde eine retrospektive Analyse von 124 Kindern mit Hörstörungen durchgeführt, die im Zeitraum von 12 Monaten im Alter zwischen 6 Monaten und 17 Jahren (Median 5,5 Jahre) im Rahmen einer Hördiagnostik neurootologisch untersucht wurden. Die Untersuchung umfasste eine ausführliche Anamnese, eine Leuchtbrillentestung, die Ableitung zervikaler und okulärer vestibulär evozierter myogener Potenziale (cVEMP, oVEMP) sowie einen Video-Kopfimpulstest (vKIT).

**Ergebnisse:**

Bei 52 % der 122 apparativ untersuchten schwerhörigen Kinder wurden vestibuläre Auffälligkeiten festgestellt, darunter 4,5 % mit isolierten pathologischen Bogengangfunktionen (5/110), 34,5 % mit gestörten Otolithenfunktionen (38/110) und 20,4 % mit kombinierten Dysfunktionen (20/98). Bei zwei Kindern war eine apparative Diagnostik nicht möglich. Bei sieben schwerhörigen Kindern wurde erstmals eine bilaterale Vestibulopathie diagnostiziert.

**Schlussfolgerung:**

Diese Arbeit zeigt, dass die Gleichgewichtsdiagnostik bei Kindern mit Hörstörungen aller Altersklassen möglich ist und empfiehlt deren routinemäßige Durchführung. Künftige Langzeitstudien sind erforderlich, um die langfristigen Folgen vestibulärer Dysfunktionen und die Effektivität therapeutischer Maßnahmen besser zu verstehen.

## Abstract (en)

**Background:**

Children with hearing impairment are at an increased risk of vestibular disorders. Due to the diagnostic challenges associated with pediatric populations, corresponding assessments are rarely performed, resulting in a lack of prevalence data in Germany. The findings, however, could have significant implications for subsequent diagnostic processes, therapeutic interventions, and preventive measures.

**Objective:**

This study investigated the feasibility of balance diagnostics for children of all age groups and emphasizes the necessity of their routine application in children with hearing impairment. Additionally, the prevalence of balance disorders in this population was assessed.

**Materials and methods:**

A retrospective analysis was performed on 124 children with hearing disorders who had undergone neurootologic assessment over a 12-month period. The children were aged between 6 months and 17 years (median age 5.5 years). Diagnostic workup included a detailed medical history, examination with Frenzel glasses, cervical and ocular vestibular evoked myogenic potentials (cVEMP, oVEMP), and a video head impulse test (vHIT).

**Results:**

Vestibular dysfunctions were identified in 52% of the 122 children with hearing impairment who underwent instrumental assessment, with 4.5% exhibiting isolated semicircular canal abnormalities (5/110), 34.5% showing otolith dysfunctions (38/110), and 20.4% presenting with combined dysfunctions (20/98). Instrumental diagnostics could not be performed in two children. Additionally, a diagnosis of bilateral vestibulopathy was established for the first time in seven children.

**Conclusion:**

This study demonstrates that vestibular diagnostics are feasible in children with hearing impairment of all age groups and advocates for their routine integration into clinical practice. Future longitudinal studies are needed to better understand the long-term consequences of vestibular dysfunctions and the effectiveness of therapeutic interventions.

Kinder mit Schwerhörigkeit und Gleichgewichtsstörungen stehen vor besonderen Herausforderungen, die ihre sprachliche, motorische und kognitive Entwicklung beeinträchtigen können. Während das Neugeborenen-Hörscreening als Standard zur Früherkennung von Hörstörungen etabliert ist, bleibt eine vestibuläre Dysfunktion häufig unerkannt. Diese Arbeit zeigt, dass eine umfassende Gleichgewichtsdiagnostik bei Kindern aller Altersstufen durchführbar ist – insbesondere bei Kindern mit Schwerhörigkeit sollte sie routinemäßig erfolgen, um Entwicklungsverzögerungen frühzeitig zu erkennen und gezielt zu therapieren.

## Gleichgewichtsdiagnostik bei Kindern als Ziel

Kinder mit Schwerhörigkeiten und Gleichgewichtsstörungen sind schon allein aufgrund ihrer Hörstörung Bedingungen ausgesetzt, die ihre sprachliche und allgemeine Entwicklung beeinträchtigen können. Dies kann sich negativ auf ihre Bildungschancen und Lebensqualität auswirken [17, 22]. Das Gleichgewichtssystem hingegen spielt eine entscheidende Rolle bei der Koordination von Bewegungen und der Aufrechterhaltung der Rumpfstabilität. Dies gilt im Alltag und besonders während körperlicher Aktivitäten. Bei Kindern mit Schwerhörigkeit und Gleichgewichtsstörungen können daher Verzögerungen in der motorischen Entwicklung auftreten [27]. Eine weitere wichtige Funktion des vestibulären Systems ist die Steuerung des vestibulookulären Reflexes (VOR), der eine Blickstabilisierung bei Bewegungen ermöglicht. Studien zeigen, dass eine durch vestibuläre Hypofunktion bedingte Blickinstabilität die Lesefähigkeit bei jungen Kindern beeinträchtigt und signifikant mit Lernschwierigkeiten assoziiert sein kann [4, 26].

Während sich für die Früherkennung von Schwerhörigkeiten das Neugeborenen-Hörscreening als Standard etabliert hat [6], werden Gleichgewichtsstörungen bei Kindern in Deutschland nur sehr selten diagnostiziert. Dabei haben Kinder mit einer Innenschwerhörigkeit ein höheres Risiko für Funktionsstörungen der Gleichgewichtsorgane [8, 32], auch bei Kindern mit einem einseitigen hochgradigen Hörverlust [28]. Ein Drittel aller Gleichgewichtsstörungen bei Kindern haben einen vestibulären Ursprung, bei Kindern mit einer Schwerhörigkeit steigt dieser Anteil auf über 50 % an [38]. Ein strukturierter Ansatz zur Erfassung vestibulärer Defizite wird im Rahmen des belgischen Projekts „Vestibular Infant Screening-Flanders“ verfolgt. Dort konnte gezeigt werden, dass ein standardisiertes vestibuläres Screening mittels zervikal vestibulär evozierter myogener Potenziale (cVEMP) bei hörgeschädigten Säuglingen im Alter von sechs Monaten sowohl praktikabel ist als auch eine frühzeitige Erkennung vestibulärer Dysfunktionen ermöglicht [18].

Da Gleichgewichtsstörungen v. a. bei einseitigen Läsionen auch durch zentrale Mechanismen kompensiert werden können [30] und betroffene Kinder dadurch im Verlauf motorisch zu ihren altersgerecht entwickelten Peers aufschließen können [15], bleiben sie häufig lange unentdeckt. Dabei können Gleichgewichtsstörungen ebenfalls erhebliche Auswirkungen auf die Lebensqualität der betroffenen Kinder haben [7]. Eine frühzeitige Diagnose einer vestibulären Dysfunktion ist entscheidend, um die Auswirkungen auf die motorische und posturale Entwicklung zu minimieren, die sich sonst in deren Folge negativ auf die kognitive Entwicklung wie auch die Orientierung im Raum und die Schreibfähigkeiten auswirken könnten [35].

Das Hörorgan ist sowohl anatomisch als auch funktionell eng mit dem Gleichgewichtsorgan verknüpft. Dies erklärt die komplexe Wechselwirkung zwischen Schwerhörigkeiten und Gleichgewichtsstörungen. So führen einige Ursachen von Innenohrschwerhörigkeiten, wie bestimmte genetische Syndrome (beispielsweise das Usher- oder Waardenburg-Syndrom) sowie Fehlbildungen, auch direkt zu Beeinträchtigungen des Gleichgewichtssystems [5, 16, 34].

Um betroffenen Kindern die bestmögliche Unterstützung zu ermöglichen, ist eine ganzheitliche Bewertung und Behandlung anzustreben. Da eine Gleichgewichtsdiagnostik bei Kindern teilweise aufwendig sein kann (und einige Untersuchungsmethoden eine aktive Mitarbeit fordern), werden solche Untersuchungen bei Kindern nur selten durchgeführt. Ziel dieser Arbeit war es aufzuzeigen, dass eine Gleichgewichtsdiagnostik für Kinder aller Altersklassen möglich ist und dass sie insbesondere bei Kindern mit Schwerhörigkeit in Deutschland routinemäßig durchgeführt werden sollte. Zudem wurde die Prävalenz von Gleichgewichtsstörungen bei Kindern mit Schwerhörigkeit erhoben.

## Methoden

### Studiendesign

Die vorliegende Studie wurde retrospektiv anhand bereits erhobener, pseudonymisierter Daten unserer klinischen Routine durchgeführt. Die pädaudiologische Untersuchung wurde entsprechend dem Lebens- und Entwicklungsalter des Kindes durchgeführt und umfasste sowohl subjektive als auch objektive Verfahren. Die Hörstörung wurde für jedes Ohr seitengetrennt dokumentiert. Bei einigen der Kinder fand die neurootologische Untersuchung bei Erstdiagnostik der Hörstörung statt, während die Erstversorgung zeitgleich eingeleitet wurde. Andere Kinder waren bereits mit ein- oder beidseitigen Hörhilfen, wie Hörgerät oder Cochleaimplantat (CI), versorgt oder sollten diese künftig erhalten.

Neben einer fundierten Anamnese zur motorischen Entwicklung wurde eine spontane Beobachtung durchgeführt, bei der Fähigkeiten wie Stehen, Hinsetzen, Aufstehen, Umdrehen, Gehen und Rennen beurteilt wurden. Klinisch wurde zudem eine Leuchtbrillentestung zur Untersuchung von Spontan- und Provokationsnystagmus durchgeführt.

### Vestibuläre Diagnostik, Otholithenfunktionstestung

Die Sacculusfunktion wurde mittels zervikal vestibulär evozierter myogener Potenziale (cVEMP) erfasst, deren Ableitung bereits ab den ersten Lebensmonaten möglich ist. Die Utrikulusfunktion wurde über okulär vestibulär evozierte myogene Potenziale (oVEMP) überprüft. Obwohl die Literatur eine Ableitung der oVEMP überwiegend erst ab dem 2. Lebensjahr beschreibt [39], konnte sie in unserer klinischen Erfahrung auch bei jüngeren Kindern erfolgreich durchgeführt werden.

Die Stimulation erfolgte über ein Eclipse-System (Interacoustics, Middelfart, Dänemark) mit einem Knochenleitungshörer B81, wodurch die Untersuchung unabhängig von intakten Mittelohrverhältnissen möglich war. Als Stimulus diente ein 500-Hz-Toneburst (0–1–0). Pro Messung wurden 60–100 Mittelungen durchgeführt; es wurden jeweils zwei reproduzierbare Kurven gefordert. Die Knochenleitungssonde wurde auf dem Mastoid der zu prüfenden Seite platziert. Bei auffälligen Asymmetrien erfolgten eine erneute Positionierung und Wiederholung der Messung.

### Zervikale vestibulär evozierte myogene Potenziale

Für die cVEMP-Ableitung wurde die aktive Elektrode jeweils über dem M. sternocleidomastoideus angebracht, die Referenzelektrode mittig über dem Sternum und die Erdungselektrode auf der Stirn. Die Muskelvorspannung wurde mit dem Messsystem in Echtzeit visualisiert; es wurde eine Untergrenze von 100 µV und eine Obergrenze von 280 µV gewählt. Die Amplituden wurden, wie vom System vorgesehen, automatisch auf die Vorspannung korrigiert. Die Kopfposition wurde je nach Alter und Kooperationsfähigkeit angepasst: Bei älteren Kindern erfolgte die Kopfwendung aktiv nach rechts oder links, alternativ in Richtung des Elternteils oder eines Bildschirms. Bei Säuglingen und Kleinkindern, die den Kopf bereits selbst halten konnten, saßen die Kinder auf dem Schoß der Eltern, einander gegenüber. Durch leichtes Zurücklehnen neigte sich der Kopf automatisch nach vorne, wodurch eine stabile Muskelvorspannung erreicht wurde.

### Okuläre vestibulär evozierte myogene Potenziale

Für die oVEMP-Ableitung befand sich die aktive Elektrode am infraorbitalen Rand des dem Stimulus kontralateralen Auges, möglichst nahe am M. obliquus inferior. Die Referenzelektrode wurde entweder direkt unterhalb der aktiven Elektrode oder auf der Stirn angebracht. Die Erdungselektrode befand sich stets auf der Stirn, oberhalb der Referenzelektrode. Konnte ein Kind den Blick nach oben nicht halten, wurde die Messung neu gestartet und das Kind mit einem neuen, meist visuellen Stimulus abgelenkt.

Bestimmt wurden die Amplituden im typischen Zeitfenster für die cVEMP p1-n1 mit 13–23 ms und für die oVEMP n1-p1 mit 10–15 ms [23]. Als pathologisch wurde eine Amplituden-Asymmetrie (Asymmetrie-Ratio) von ≥ 0,3 gewertet; zusätzlich wurde ein fehlendes Signal ebenfalls als pathologisch eingestuft.

**Abb. 1:**
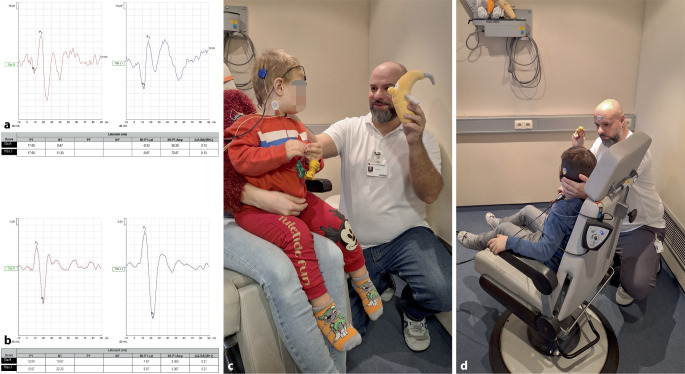
Messung vestibulär evozierter myogener Potenziale (VEMP) bei Kindern. Abbildung (**a**) zeigt die Ableitung okulärer vestibulär evozierter myogener Potenziale (oVEMP) eines 2‑jährigen Kindes im typischen Zeitfenster mit annähernd symmetrischer Amplitudenhöhe. Die Amplituden sind erhöht, wie häufig bei Kindern beobachtet, ohne klinische oder anamnestische Hinweise auf ein „drittes Fenster“. In (**b**) ist die Ableitung zervikaler vestibulär evozierter myogener Potenziale (cVEMP) eines knapp 1‑jährigen Kindes dargestellt; die cVEMP-Kurven sind skaliert und EMG-korrigiert, die Asymmetrie liegt im Normbereich. (**c**, **d**) Klinisches Setting der cVEMP-Messung bei einem 2‑jährigen (**c**) bzw. 5‑jährigen Kind (**d**); dargestellt sind kindgerechte Strategien zur Förderung der Mitarbeit während der Untersuchung

Die Abb. 1c, d zeigen exemplarisch die VEMP-Messung bei Kindern.

### Vestibuläre Diagnostik, Bogengangfunktionstestung

Zusätzlich wurde ein Video-Kopfimpulstest (vKIT) durchgeführt. In den ersten 7 Monaten des Untersuchungszeitraums von Januar bis Juli 2023 erfolgte die Messung mit der EyeSeeCam-Videobrille (Interacoustics. Middelfart, Dänemark; Version 1.3), seit dem 01.08.2023 wurde die Videobrille Synapsys VHIT (Inventis, Marseille, Frankreich. Ulmer II Version 3.4.1) verwendet. Damit wurde die Untersuchung kabellos durchgeführt. Immer getestet wurden die lateralen Bogengänge und je nach Mitarbeit auch die vorderen und hinteren (Left Anterior Right Posterior, LARP, und Right Anterior Left Posterior,  RALP). Es wurden dabei mindestens 5 Messungen pro Seite durchgeführt. Als pathologisch gewertet wurden ein Gain ≤ 0,7 [11, 29] und gleichzeitig vorhandene kumulierte Rückstellsakkaden.

Es wurde der vom Hersteller empfohlene Aufbau verwendet, mit einem Abstand von 90 cm zwischen Kopf und Kamera sowie weiteren 90 cm zwischen Kamera und Wand mit dem Blickziel. Als Fixationspunkt diente entweder der standardisierte Zielpunkt des Herstellers oder ein altersgerechter Reiz (z. B. leuchtender Ball, Smartphone, Untersucher mit roter Clownsnase oder Handpuppe). Kleine Kinder saßen je nach Alter und Kooperationsfähigkeit allein oder auf dem Schoß eines Elternteils, sodass die Untersucherin bzw. der Untersucher hinter dem Kind stehen konnte.

**Abb. 2:**
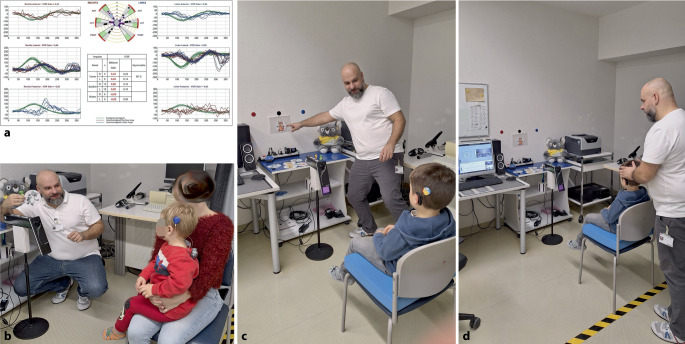
Video-Kopfimpulstest (vKIT). Unter (**a**) ist ein exemplarischer Befund eines vKIT bei einem 9‑jährigen Kind dargestellt. Es zeigt sich hier eine bilaterale Vestibulopathie mit pathologischen Befunden der horizontalen Bogengänge beidseits mit Gain ≤ 0,7 und Rückstellsakkaden. Die Abbildungen (**b**)–(**d**) zeigen das klinische Setting der vKIT-Untersuchung bei einem 2‑jährigen Kind (**b**) sowie bei einem 5‑jährigen Kind (**c**, **d**). Die Fotos illustrieren zudem, wie Kinder spielerisch zur aktiven Mitarbeit im Rahmen der Untersuchung motiviert werden können

**Abb. 3:**
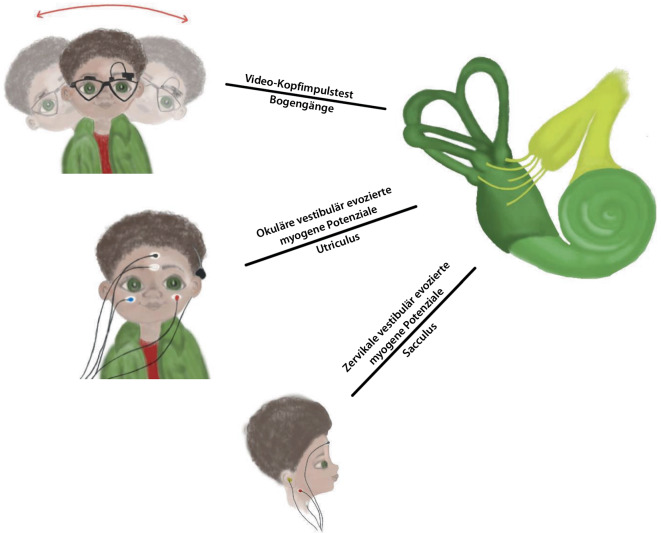
Schematische Darstellung des Versuchsdesigns mit Video-Kopfimpulstest (vKIT) sowie Messung vestibulär evozierter myogener Potenziale (VEMP; cVEMP: zervikale VEMP; oVEMP: okuläre VEMP). Dargestellt sind die Elektrodenpositionen der VEMP-Messung und die Durchführung des vKITs (nicht maßstabsgetreu)

Eine vKIT-Untersuchung bei Kindern ist in Abb. 2b–d dargestellt; das Versuchsdesign für die vestibuläre Diagnostik ist schematisch in Abb. 3 gezeigt.

Der durchschnittliche Zeitaufwand betrug 30 min. Die Durchführung erfolgte je nach Aufwand durch 1–2 neurootologisch geschulte Audiologieassistent*innen. Die retrospektive Datenauswertung wurde durch zwei ärztliche Mitarbeiter*innen vorgenommen.

### Ethik

Das Votum wurde von der zuständigen Ethikkommission der Landesärztekammer Rheinland-Pfalz (Aktenzeichen 2024-17755-retrospektiv) eingeholt.

### Datenauswertung

Die statistische Analyse erfolgte mit jamovi [21]. Zur statistischen Auswertung wurden Prävalenzen berechnet sowie Korrelationen mittels Pearson‑r und Chi-Quadrat-Test analysiert. Als Signifikanzniveau wurde *p* < 0,05 festgelegt; die Ergebnisse wurden mit folgendem Hinweis gekennzeichnet: * *p* < 0,05; ** *p* < 0,01; *** *p* < 0,001.

## Ergebnisse

Es wurden innerhalb eines Zeitraums von 12 Monaten insgesamt 124 schwerhörige Kinder an der Hals-Nasen-Ohren-Klinik, Abteilung Kommunikationsstörung der Universitätsmedizin Mainz, untersucht. Von diesen Kindern wiesen 92 eine Innenohrschwerhörigkeit (74,2 %) sowie 17 eine Schallleitungsschwerhörigkeit (13,7 %) auf, und bei 15 Kindern lag eine kombinierte Schwerhörigkeit (12,1 %) vor. Bei fünf Kindern lagen hierbei unterschiedliche Schwerhörigkeitsarten auf dem rechten und linken Ohr vor, und bei einem Kind wurde differenzialdiagnostisch eine auditorische Synaptopathie‑/Neuropathie diskutiert. Der Grad der Schwerhörigkeit wurde in Anlehnung an die in Deutschland gebräuchliche Skala nach Röser 1973 angegeben [3]. Die genaue Verteilung ist in Tab. 1 dargestellt. Bei 33 der Kinder (27 %) lag eine einseitige Schwerhörigkeit vor, wobei bei 15 dieser Kinder eine einseitige Taubheit bzw. eine an Taubheit grenzende Schwerhörigkeit mit normalem Hörvermögen der Gegenseite festgestellt wurde. Insgesamt 25 Kinder waren bereits mit einem CI versorgt: 13 Kinder waren dabei beidseits mit einem CI versorgt, 5 auf der rechten Seite und 7 Kinder waren mit einem CI links versorgt. Ein Kind war mit einem OSIA-Hörsystem versorgt.

**Tab. 1:** Prävalenz von Schwerhörigkeit nach Röser 1973, Gewichtungsmerkmale für rechtes und linkes Ohr.

Hörverlust (Röser 1973 in %)	Interpretation	Rechtes Ohr	Linkes Ohr
0–9	Normales Hörvermögen	10 % (12/124)	13 % (16/124)
10–19	Annähernd normales Hörvermögen	4 % (5/124)	4 % (5/124)
20–29	Annähernd geringgradige Schwerhörigkeit	3 % (4/124)	6 % (7/124)
30–39	Geringgradige Schwerhörigkeit	7 % (9/124)	6 % (8/124)
40–49	Gering- bis mittelgradige Schwerhörigkeit	9 % (11/124)	6 % (7/124)
50–59	Mittelgradige Schwerhörigkeit	10 % (12/124)	8 % (10/124)
60–69	Mittel- bis hochgradige Schwerhörigkeit	4 % (5/124)	6 % (7/124)
70–79	Hochgradige Schwerhörigkeit	5 % (6/124)	7 % (9/124)
80–89	Hochgradige- bis an Taubheit grenzende Schwerhörigkeit	6 % (7/124)	3 % (4/124)
90–99	An Taubheit grenzende Schwerhörigkeit	14 % (17/124)	13 % (16/124)
100	Taubheit	29 % (36/124)	28 % (35/124)

**Abb. 4:**
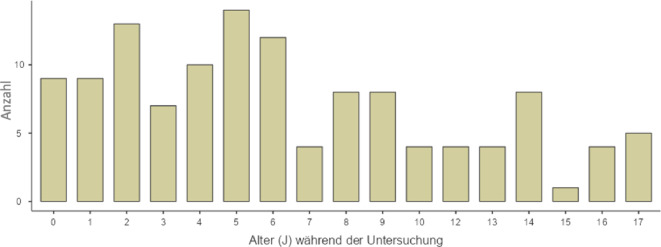
Häufigkeitsverteilung des Alters zum Zeitpunkt der Untersuchung. Die *Balken* zeigen die absolute Häufigkeit pro Altersintervall. Das Alter der Kinder und Jugendlichen lag zwischen 6 Monaten und 17 Jahren, mit einem Median von 5,5 Jahren (*J*; Standardabweichung, SD: 4,9)

Es waren 61 Mädchen und 63 Jungen. Das Alter der Kinder und Jugendlichen lag zwischen 6 Monaten und 17 Jahren, mit einem Median von 5,5 Jahren (Standardabweichung, SD: 4,9; Abb. 4).

Bei allen 124 schwerhörigen Kindern erfolgte mindestens eine orientierende Funktionseinschätzung im Rahmen der klinischen Gleichgewichtsdiagnostik. Bei der apparativen Testung wurden bei 110 Kinder VEMP abgeleitet, und bei 110 Kindern wurde ein vKIT durchgeführt. Beide Untersuchungen erfolgten bei 98 Kindern. Bei 2 Kindern konnte keine apparative Untersuchung durchgeführt werden, da diese nicht toleriert wurde. Dabei handelte es sich um ein knapp 7 Jahre altes Kind mit schwerster geistiger Entwicklungsretardierung und um ein knapp 3 Jahre altes Kind mit motorischer Unruhe. Bei diesen Kindern konnte klinisch ein Kopfimpulstest durchgeführt werden, welcher nicht in die Auswertung mitaufgenommen wurde, um eine sichere Vergleichbarkeit der Methoden zu gewährleisten.

**Abb. 5:**
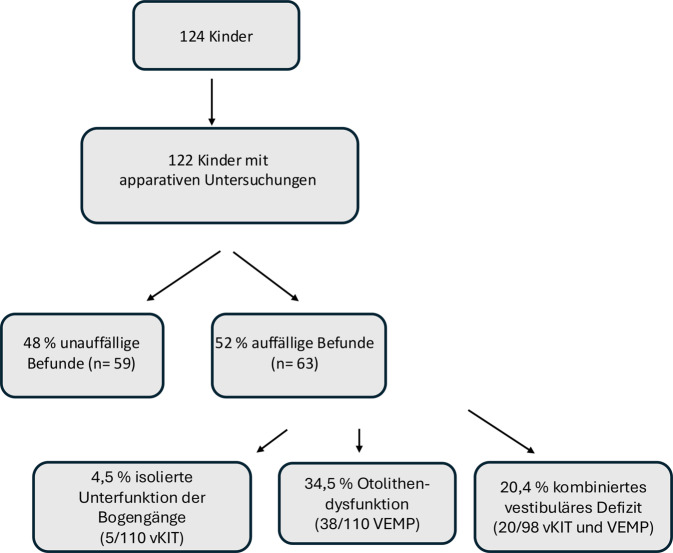
Übersicht der vestibulären Untersuchungsergebnisse bei 122 schwerhörigen Kindern. Dargestellt sind die Verteilungen unauffälliger Befunde, isolierter Unterfunktion der Bogengänge (Video-Kopf-Impulstest, vKIT), isolierte Otolithendysfunktion (vestibulär evozierte myogene Potenziale, VEMP) sowie kombinierter vestibulärer Defizite (vKIT und VEMP)

Die Kinder wurden in zwei Gruppen eingeteilt: solche ohne vestibuläre Auffälligkeiten und solche mit vestibulären Auffälligkeiten, d. h. mit Nachweis von funktionellen Defiziten in den Bogengängen oder Otolithenorganen. Hierzu zählen pathologische Gain-Werte im vKIT oder fehlende bzw. asymmetrische VEMP-Potenziale, bewertet im typischen Zeitfenster. Letztere Gruppe wurde weiter unterteilt in eine Gruppe mit einer Auffälligkeit entweder bei Ableitung der VEMP oder dem vKIT und eine Gruppe mit Auffälligkeiten in beiden Untersuchungen. Bei 63 der 122 apparativ untersuchten Kinder wurden Auffälligkeiten festgestellt (52 %) (Abb. 5).

### Pathologische Bogengangfunktion

Pathologische Befunde im Bereich der horizontalen Bogengänge wurden bei 20 von 110 Kindern (18,2 %) festgestellt. Die Befunde waren dabei in 3 Fällen rechtsseitig (2,7 %), in 10 Fällen linksseitig (9,1 %) und in 7 Fällen beidseits (6,6 %) auffällig.

Bei der vertikalen Bogengangtestung (RA/RP und LA/LP, *n* = 86) zeigten 8 Kinder (9,3 %) pathologische Befunde: rechtsseitig in 2 Fällen (2,3 %), linksseitig in 3 Fällen (3,5 %) sowie beidseits in ebenfalls 3 Fällen (3,5 %).

Bei fünf der 110 durchgeführten vKIT (4,5 %) handelte es sich um isolierte pathologische Befunde der Bogengänge.

Ein exemplarischer Befund ist in Abb. 2a dargestellt.

### Gestörte Otolithenfunktion

Bei Ableitung der cVEMP zeigte sich bei 40 von 110 Kindern (36,4 %) eine gestörte otolithäre Funktion: rechtsseitig bei 12 (10,9 %), linksseitig bei 19 (17,3 %) und beidseits (d. h. fehlende VEMP-Potenziale) bei 9 Kindern (8,2 %).

Bei den oVEMP fanden sich bei 41 von 100 Kindern (41 %) pathologische Befunde: rechtsseitig bei 14 (14 %), linksseitig bei 18 (18 %) und beidseits bei 9 Kindern (9 %).

Bei insgesamt 38 der 110 untersuchten Kinder (34,5 %) konnte eine isolierte pathologische Otolithenfunktion (cVEMP oder/und oVEMP) nachgewiesen werden.

Bei 14 Kindern unter zwei Jahren lagen oVEMP-Ergebnisse vor; davon zeigten 10 unauffällige und 4 pathologische Befunde. Bei weiteren 4 Kindern unter zwei Jahren wurde die oVEMP-Untersuchung nicht durchgeführt.

Eine exemplarische Darstellung der Befunde ist in Abb. 1a, b zu sehen.

### Kombinierte pathologische Befunde

Bei 14 von 98 Kindern (14,3 %) zeigte sich eine kombinierte, pathologische Bogengang- und gestörte Sacculusfunktion (Auffälligkeiten bei Ableitung der cVEMP und vKIT). Dreiundzwanzig von 100 Kindern (23 %) hatten sowohl bei den cVEMP als auch bei den oVEMP auffällige Ergebnisse.

Insgesamt wiesen 20 Kinder (20,4 %) kombinierte pathologische Befunde im vKIT und in mindestens einer VEMP-Messung (cVEMP oder/und oVEMP) auf.

Eine genaue Auflistung liefert Tab. 2.

**Tab. 2:** Ergebnisse der vestibulären Funktionsdiagnostik bei Kindern mit Hörstörung: Video-Kopfimpulstest (vKIT) und vestibulär evozierte myogene Potenziale (VEMP).

Testmethode	Befund	Anzahl	Anteil an Gesamtzahl (in %)
*vKIT horizontal (n* *=* *110)*	Unauffällig	90	81,8
	Rechts auffällig	3	2,7
	Links auffällig	10	9,1
	Auffällig beidseits	7	6,6
*vKIT RA/RP und LA/LP (n* *=* *86)*			
–	Unauffällig	78	90,7
RA/RP	Rechts auffällig	2	2,3
LA/LP	Links auffällig	3	3,5
–	Auffällig beidseits	3	3,5
*cVEMP (n* *=* *110)*	cVEMP unauffällig	70	63,6
	cVEMP rechts auffällig	12	10,9
	cVEMP links auffällig	19	17,3
	cVEMP auffällig beidseits	9	8,2
*oVEMP (n* *=* *100)*	Unauffällig	59	59
	Rechts auffällig	14	14
	Links auffällig	18	18
	Auffällig beidseits	9	9
*Kombinierte Befunde*			
(*n* = 98)	cVEMP und vKIT auffällig	14	14,3
(*n* = 98)	vKIT und VEMP (cVEMP oder/und oVEMP)	20	20,4
(*n* = 100)	cVEMP und oVEMP auffällig	23	23

*vKIT* Video-Kopfimpulstest; *horizontal* Testung der horizontalen Bogengänge; *RA/RP* Testung des rechten anterioren (RA) und rechten posterioren (RP) Bogengangs; *LA/LP* Testung des linken anterioren (LA) und linken posterioren (LP) Bogengangs, *VEMP* vestibulär evozierte myogene Potenziale (*cVEMP* zervikal; *oVEMP* okulär)

Es fand sich ein schwacher Zusammenhang zwischen dem Grad der Schwerhörigkeit rechts und einer pathologischen Bogengangfunktion (lateral Pearson‑r 0,221; *p* = 0,02; anterior und posterior Pearson‑r 0,239; *p* = 0,03) sowie einer Dysfunktion des Sacculus (Pearson‑r 0,207; *p* = 0,03). Ähnliche Befunde fanden sich für die linke Seite (lateraler Bogengang Pearson‑r 0,215; *p* = 0,03; cVEMP Pearson‑r 0,227; *p* = 0,02). Es zeigte sich kein signifikanter Zusammenhang zwischen dem Grad der Schwerhörigkeit links und der Funktion der vertikalen Bogengänge derselben Seite (anterior und posterior; Pearson‑r 0,156; *p* = 0,149). Ebenso bestand kein signifikanter Zusammenhang zwischen dem jeweiligen Grad der Schwerhörigkeit und einer Utrikulusdysfunktion (rechts: oVEMP Pearson‑r 0,168; *p* = 0,095; links: oVEMP Pearson‑r 0,103; *p* = 0,306; Tab. 3).

**Tab. 3:** Korrelationskoeffizienten (Pearson-r) zwischen den pathologischen Befunden (ja/nein) verschiedener vestibulärer Testverfahren (vKIT = Video-Kopfimpulstest; cVEMP = zervikale vestibulär evozierte myogene Potenziale; oVEMP = okuläre vestibulär evozierte myogene Potenziale) und dem Ausmaß der Hörminderung (Grad der Schwerhörigkeit nach Röser 1973, rechts und links). Es werden jeweils der Korrelationskoeffizient sowie der zugehörige *p*-Wert angegeben.

		Schwerhörigkeit rechts	Schwerhörigkeit links
vKIT	Pearson‑r	0,221*	0,215*
lateraler Bogengang	*p*-Wert	0,020	0,025
vKIT	Pearson‑r	0,239*	–
RA/RP	*p*-Wert	0,028	–
vKIT	Pearson‑r	–	0,156
LA/LP	*p*-Wert	–	0,149
cVEMP	Pearson‑r	0,207*	0,227*
	*p*-Wert	0,032	0,018
oVEMP	Pearson‑r	0,168	0,103
	*p*-Wert	0,095	0,306

Hinweis. * *p* < 0,05; ** *p* < 0,01; *** *p* < 0,001

Von den 25 CI-versorgten Kindern zeigten 17 Kinder auffällige Befunde in der Gleichgewichtsdiagnostik. Pathologische Befunde im Bereich der Bogengänge traten bei 9 Kindern auf, davon 4 bei der Testung der linken Bogengänge und 5 mit beidseitigen Auffälligkeiten. Bei der Ableitung der VEMP zeigte sich ein auffälliger Befund rechts bei einem Kind, links bei 8 Kindern und beidseits bei 6 Kindern. Ein signifikanter Zusammenhang zwischen der CI-Versorgung und einem pathologischen Gleichgewichtsbefund (auffällig vs. unauffällig) konnte nicht nachgewiesen werden (Chi-Quadrat-Test, χ^2^ = 2,60; *p* = 0,107).

Bei sieben schwerhörigen Kindern wurde die Erstdiagnose einer bilateralen Vestibulopathie gestellt, dabei war der vKIT bei Testung der horizontalen Bogengänge beidseits auffällig. Bei 3 der Kinder konnten zusätzlich die vorderen und hinteren Bogengänge getestet werden, welche ebenfalls auffällig waren. Eine beispielhafte Darstellung liefert Abb. 2a. Von diesen sieben Kindern wiesen sechs eine beidseitige Hörrestigkeit mit einem Hörverlust von > 99 % nach Röser (1973) auf. Ein Kind hatte beidseits eine an Taubheit grenzende Schwerhörigkeit (nach Röser 1973). Bei drei Kindern war eine syndromale Ursache der Schwerhörigkeit bekannt, darunter das Usher-Syndrom. Bei einem weiteren Kind bestand der Verdacht auf eine syndromale Erkrankung, und ein Kind hatte eine genetisch gesicherte, nichtsyndromale Hörstörung. Bei zwei Kindern blieb die Ursache der Schwerhörigkeit auch nach genetischer Testung bis einschließlich zur dritten Diagnosestufe unklar. Bildmorphologisch zeigte sich bei einem Kind zusätzlich eine komplexe Innenohrfehlbildung mit unter anderem beidseits dysplastischen Bogengängen. Zum Untersuchungszeitpunkt waren sechs Kinder mit einem CI versorgt, davon drei unilateral. Ein 2‑jähriges Kind konnte zum Zeitpunkt der Datenerhebung noch nicht laufen, ein weiteres Kind hatte laut Anamnese erst im Alter von drei Jahren das Laufen erlernt. Bei zwei Kindern wurde über wiederholte Stürze berichtet. Ein Kind wies schulische Schwierigkeiten mit deutlichen Beeinträchtigungen im Lese- und Rechtschreiberwerb auf.

## Diskussion

In unserer Kohorte zeigte sich eine Prävalenz von 52 % für vestibuläre Auffälligkeiten bei schwerhörigen Kindern. Diese liegt deutlich über der von Saniasiaya et al. angegebenen Prävalenz von 30,4 % [25]. Eine kürzlich veröffentliche Arbeit von Wiener-Vacher et al. untersuchte eine Kohorte von Kindern mit hochgradiger Schwerhörigkeit und zeigte, dass bei 44,4 % der Kinder eine teilweise beeinträchtigte vestibuläre Funktion vorlag. Bei 5,7 % der Kinder wurde eine bilaterale Vestibulopathie diagnostiziert. Genetisch bedingte, syndromale Hörstörungen sowie infektiös (hauptsächlich durch CMV) bedingte Hörstörungen waren häufiger mit einer bilateralen Vestibulopathie assoziiert als andere Ursachen der Hörstörungen. Zudem stieg das Alter, in dem die Meilensteine der Entwicklung erreicht wurden, mit dem Grad der vestibulären Beeinträchtigung [36]. Eine große US-amerikanische retrospektive Analyse von 2010 mit über 560.000 pädiatrischen Patientenkontakten zeigte insgesamt eine niedrige Prävalenz von Gleichgewichtsstörungen (0,45 %), jedoch deutliche Assoziationen zu sensorineuralem Hörverlust [19].

Die Verwendung von VEMP und Video-Kopfimpulstests als Diagnosetools ist weit verbreitet und gilt als Goldstandard in der Diagnostik der vestibulären Funktion [1, 24]. Diese apparativen Testverfahren haben sich als effektiv erwiesen. Eine Studie belegt die Anwendbarkeit des vKIT bei Kindern generell [12], während eine andere besonders den Nutzen und die Durchführbarkeit der cVEMP-Ableitung bei Kindern mit Hörstörungen hervorhebt [20]. Bisher wurden jedoch selten Kinder unter drei Jahren in solche Studien einbezogen. In unserer Kohorte waren 38 der Kinder 3 Jahre und jünger. Lediglich bei zwei der 124 Kinder war keine apparative Testung möglich. Diese Kinder wiesen zusätzlich eine schwere geistige Entwicklungsverzögerung auf, bzw. bei dem knapp dreijährigen Kind lag eine motorische Unruhe vor, und die Untersuchung wurde zu diesem Zeitpunkt verweigert. Bei beiden Kindern war dennoch eine orientierende klinische Einschätzung mit dem Kopfimpulstest möglich, die jedoch nicht in die Auswertung mit einbezogen wurde.

Die Durchführung apparativer vestibulärer Testverfahren im frühen Kindesalter ist mit spezifischen Herausforderungen verbunden. Begrenzte Kooperationsfähigkeit, motorische Unruhe und eine altersbedingt geringe Aufmerksamkeitsspanne stellen potenzielle Fehlerquellen dar. Testspezifisch können beim vKIT ein Schließen der Augen oder das Nichtfixieren des Blickziels zu fehlerhaften Ergebnissen führen. Bei oVEMP sind Schwierigkeiten, den Blick nach oben zu halten, ebenso wie starke Gegenwehr oder Anspannung häufige Ursachen für Artefakte. Bei cVEMP führen ungleichmäßige Muskelvorspannung und eingeschränkte Mitarbeit zu variablen Ergebnissen. Eine standardisierte Testdurchführung sowie die Erfahrung der Untersuchenden sind entscheidend, um valide und klinisch verwertbare Resultate zu erzielen. Falls eine Testung nicht möglich ist, kann – abhängig von der Fragestellung – eine Wiederholung im weiteren Verlauf sinnvoll sein.

Wie von Walther beschrieben, ist für die differenzialdiagnostische Einordnung von Schwindelsyndromen eine strukturierte Anamnese, gezielte Kommunikation sowie eine objektive apparative Diagnostik erforderlich, im Idealfall mit Erfassung aller fünf vestibulären Sensoren beidseits [33], auch wenn sich diese Empfehlungen nicht explizit auf Kinder beziehen.

Darüber hinaus weisen Hülse et al. darauf hin, dass peripher-vestibuläre Störungen im Kindesalter vermutlich häufig unterdiagnostiziert werden. Unabhängig von einer Schwerhörigkeit sollten bei Kindern mit Schwindelsymptomen solche Störungen stets differenzialdiagnostisch in Betracht gezogen werden. Dies gilt besonders für sehr junge Kinder, die ihre Symptome noch nicht verbalisieren können [13].

Bei 34,5 % der Kinder konnte in unseren Untersuchungen eine Otolithendysfunktion nachgewiesen werden. Da diese in allen Altersgruppen mit geringem Aufwand erfassbar ist, sollte die Prüfung der Otolithenfunktion routinemäßig ergänzend zur Untersuchung der Bogengänge erfolgen. Eine kombinierte Dysfunktion von Bogengängen und Otolithenorganen wurde in unserer Studienkohorte bei 20,4 % der Kinder festgestellt. Da die genauen Auswirkungen der einzelnen vestibulären Ausfälle für die weitere Entwicklung noch nicht bekannt sind, sollte versucht werden, mehrere objektive Testverfahren zu kombinieren. Diese sollten sich einer fundierten Anamnese mit den Eltern und einer subjektiven Beobachtung der Kinder anschließen.

Unsere Ergebnisse decken sich mit den Befunden des systematischen Reviews von Ghai et al., der eine hohe Prävalenz otolithärer Dysfunktionen bei Kindern mit sensorineuraler Schwerhörigkeit beschreibt [10]. In zwei Studien mittlerer Qualität wurde die Prävalenz mit 17–33 % angegeben, während eine niedrigqualitative Studie sogar eine Rate von 91,3 % pathologischer Sacculus-Antworten fand. Die hohe Variabilität in der berichteten Prävalenz wird unter anderem durch Unterschiede in Methodik, Testprotokollen und Studienqualität erklärt.

Gerdsen et al. berichteten in einer Kohorte von 86 Kindern mit sensorineuraler Schwerhörigkeit von einer Prävalenz vestibulärer Hypofunktionen von 44 % und schlagen einen diagnostischen Algorithmus vor, der zunächst die Durchführung eines vKIT vorsieht, gefolgt von cVEMP bei Kindern unter vier Jahren bzw. einer kalorischen Testung bei älteren Kindern, sofern der initiale Befund unauffällig bleibt [9].

Nach unseren Daten ist dieser Ansatz zu befürworten, da sowohl bei der alleinigen Durchführung des vKIT als auch bei ausschließlicher Ableitung der cVEMP als Screeningmethode, wie beispielsweise im Rahmen des belgischen Projekts „Vestibular Infant Screening-Flanders“ [18], relevante Unterfunktionen unentdeckt bleiben können.

Vorangegangene Untersuchungen haben gezeigt, dass genetisch bedingte, syndromale Hörstörungen sowie infektiös bedingte Hörstörungen häufiger mit einer bilateralen Vestibulopathie assoziiert sind als andere Ursachen der Hörstörungen [36]. In unserer Arbeit wurde bei sieben Kindern die Erstdiagnose einer bilateralen Vestibulopathie gestellt. Durch die beidseitige Vestibulopathie und die dadurch eingeschränkte Teilhabe an sportlichen Aktivitäten Gleichaltriger steigt das Risiko für ein Übergewicht. Zusätzlich zum Bewegungsmangel kann dies Einfluss auf die sozial-emotionale Entwicklung der Kinder haben, z. B. in Form eines sozialen Rückzugs oder psychischer Probleme.

Gadsbøll et al. untersuchten Kinder mit ein- oder beidseitiger Schwerhörigkeit mittels vKIT, cVEMP und oVEMP. Es wurde gezeigt, dass Ohren mit zwei pathologischen Untersuchungsergebnissen einen signifikant schwereren Hörverlust aufwiesen als Ohren mit einer pathologischen oder normalen vestibulären Untersuchung [8]. Janky et al. identifizierten prädiktive Faktoren für ein vestibuläres Defizit, darunter war ein Hörverlust von mehr als 66 dB, verzögerte Fähigkeiten der Kinder, zu sitzen und zu laufen, und elterliche Bedenken hinsichtlich der grobmotorischen Entwicklung ihrer Kinder [14]. Dies unterstreicht noch einmal die Wichtigkeit zur Erfassung dieser Parameter durch die Eltern, um spätestens bei diesen Auffälligkeiten eine neurootologische Untersuchung zu veranlassen.

Bisherige Untersuchungen erfolgten häufig bei Kindern mit hochgradigem Hörverlust bzw. einer Taubheit. Die Untersuchungen fanden dabei vor der Evaluation einer Cochleaimplantatversorgung statt. In unserer Kohorte wurden auch Kindern mit gering- und mittelgradigem Hörverlust untersucht. Es fand sich lediglich eine schwache Korrelation zwischen dem Grad der Schwerhörigkeit und einem vestibulären Defizit (pathologische Funktion des lateralen Bogengangs sowie des Sacculus). Während frühere Studien eine Korrelation sowohl der cVEMP- als auch der oVEMP-Befunde mit dem Grad der Schwerhörigkeit beschrieben haben [2], zeigten sich in unserer Kohorte Hinweise auf einen stärkeren Zusammenhang der Schwerhörigkeit mit den cVEMP-Ergebnissen.

Die Befunde unterstützen die Durchführung einer vestibulären Diagnostik auch bei Kindern mit gering- oder mittelgradigem Hörverlust.

Die hohe Prävalenz von vestibulären Störungen bei schwerhörigen Kindern macht deutlich, dass eine Gleichgewichtsdiagnostik in dieser Risikogruppe zum Standard dazugehören sollte.

Insbesondere vor einer geplanten CI-Versorgung bei Kindern sollte eine neurootologische Diagnostik erfolgen, da sie wichtige Informationen zur Auswahl der Implantationsseite liefert und bei der Entscheidung zwischen einer simultanen oder sequenziellen beidseitigen Implantation berücksichtigt werden sollte. Wie die Studie von Thierry et al. zeigte, wies die Hälfte der Kinder präoperativ eine vestibuläre Dysfunktion auf, bei 20 % der Kinder hatte sich die vestibuläre Funktion nach CI-Implantation verschlechtert [31]. Zusätzlich zu den Einschränkungen der sprachlichen Fertigkeiten durch die Hörstörung sind die Kinder weiteren negativen Aspekten ausgesetzt. Wiener-Vacher et al. stellten dabei die Hypothese auf, dass ein Mangel an vestibulären Informationen in frühen Lebensphasen zu einer reduzierten kognitiven Leistungsfähigkeit in verschiedenen Bereichen sowie zu veränderten räumlichen kognitiven Repräsentationen führen kann, im Vergleich zu Kindern ohne vestibuläre Beeinträchtigung [37]. Darüber hinaus hat die durch vestibuläre Hypofunktion verursachte Blickinstabilität einen negativen Einfluss auf die Lesefähigkeit junger Kinder [4].

Die vorliegenden Daten decken sich mit den bisher veröffentlichen Studien und unterstützen die Annahme, dass vestibuläre Funktionsstörungen bei Kindern mit Hörstörung häufig auftreten. Da die konkreten Auswirkungen einzelner vestibulärer Ausfälle auf die kindliche Entwicklung bislang nicht ausreichend verstanden sind, erscheint der kombinierte Einsatz mehrerer objektiver Testverfahren sinnvoll, um ein möglichst umfassendes Bild der vestibulären Funktion zu erhalten. Um langfristig ein gezieltes Vorgehen empfehlen zu können, sind zudem weitere systematische Untersuchungen mit größeren Kohorten und altersbezogenen Referenzwerten erforderlich.

### Stärken und Limitationen der Studie

Eine der zentralen Stärken der vorliegenden Studie ist die Größe der Kohorte. Zudem umfasst die Stichprobe ein breites Altersspektrum, einschließlich eines hohen Anteils an Kindern unter 3 Jahren. Eine weitere Stärke der Studie liegt in der umfassenden vestibulären Diagnostik. Die Kinder wurden sowohl mittels vKIT zur Beurteilung der Bogengangfunktion als auch mittels cVEMP und oVEMP zur Überprüfung der Otolithenfunktion untersucht.

Ein wesentlicher Limitierungsfaktor der Studie ist, dass die spezifische Ursache der Hörstörung nicht in die Auswertung einbezogen wurde, da unter anderem genetische Untersuchungen bei einigen Kindern zum Zeitpunkt der Auswertung noch nicht vorlagen. Darüber hinaus sind Langzeitbeobachtungen erforderlich, um die Entwicklung von Kindern mit Hör- und Gleichgewichtsstörungen zu verfolgen und die Effektivität von Interventionen zu bewerten. Solche Studien könnten wertvolle Daten liefern, um optimale Behandlungsstrategien zu identifizieren. Unsere Ergebnisse stehen im Einklang mit der in der Literatur ausgesprochenen Empfehlung, eine kombinierte vestibuläre Diagnostik einzusetzen. Trotz dieser Übereinstimmung kann aus den vorliegenden Daten aufgrund der begrenzten Fallzahl mit kleinen Subkohorten und der Heterogenität der Befunde kein standardisierter diagnostischer Algorithmus abgeleitet werden. Hierfür sind größere und methodisch einheitlichere Studien notwendig. Zu den zentralen Limitationen der Studie gehört auch das Fehlen altersabhängiger Normwerte für die VEMP-Messungen, wodurch die Beurteilung einer möglichen beidseitigen Unterfunktion eingeschränkt ist. Zudem ist bei den CI-versorgten Kindern nicht bekannt, ob vestibuläre Unterfunktionen bereits präoperativ bestanden, sodass Rückschlüsse auf die zeitliche Entwicklung der vestibulären Defizite nur eingeschränkt möglich sind.

## Fazit für die Praxis

- Aufgrund des häufigen Auftretens peripher-vestibulärer Störungen bei schwerhörigen Kindern erscheint eine routinemäßige Diagnostik mittels gut tolerierter Tests, wie dem Video-Kopfimpulstest und der Ableitung vestibulär evozierter myogener Potenziale, sinnvoll.
- Mit entsprechender Übung lassen sich diese Untersuchungen gut in den klinischen Alltag integrieren.
- Künftige Langzeitstudien sind erforderlich, um die langfristigen Folgen vestibulärer Dysfunktionen und die Effektivität therapeutischer Maßnahmen besser zu verstehen und einen standardisierten diagnostischen Algorithmus entwickeln zu können.

## Data Availability

Die Daten, die in dieser Studie verwendet wurden, sind auf Anfrage bei der entsprechenden Autorin erhältlich.
